# Spatiotemporal Metabolome and Single‐Nucleus Transcriptome Integration Illuminates an Auxin Gradient Orchestrated by *NtTAC1* Underlying Leaf Angle Regulation in Tobacco

**DOI:** 10.1111/pbi.70672

**Published:** 2026-04-27

**Authors:** Lin Wang, Junping Gao, Chen Wang, Guoyun Xu, Zhen Ma, Shuaibin Wang, Zhaopeng Luo, Mingzhu Wu, Jianfeng Zhang, Jun Yang, Peijian Cao, Xiaodong Xie

**Affiliations:** ^1^ Beijing Life Science Academy Beijing China; ^2^ Key Laboratory of Biosynthesis and Biomanufacturing in Model Plants (Beijing Life Science Academy), Ministry of Industry and Information Technology Beijing China; ^3^ China Tobacco Gene Research Center, Zhengzhou Tobacco Research Institute of CNTC Zhengzhou China; ^4^ College of Life Sciences Henan Agricultural University Zhengzhou China; ^5^ China Tobacco Hunan Industrial Co., Ltd. Changsha China

**Keywords:** auxin redistribution, leaf angle, *NtPIN3*, *NtTAC1*, plant architecture optimization, single‐nucleus transcriptomics

## Abstract

Plant architecture is key to crop yield, with leaf angle being critical for high‐density cultivation. Although *TAC1* represents a promising regulator of leaf angle for breeding, its molecular mechanism remains poorly understood, particularly at single‐nucleus resolution. Here, we performed single‐nucleus RNA sequencing on *NtTAC1* knockdown lines exhibiting reduced leaf angle. This analysis generated a transcriptional atlas comprising 20 distinct clusters corresponding to 14 cell types and identified the endodermis as a central regulatory hub. Weighted gene co‐expression network analysis and trajectory inference revealed that the auxin transporter *NtPIN3* acts as a key downstream effector of *NtTAC1*. The two genes are co‐expressed in endodermal cells and promote their differentiation from meristematic cells. Spatial metabolomics further demonstrated that *NtTAC1* suppression elevates auxin levels and alters its spatial distribution, resulting in asymmetric auxin accumulation preferentially in the abaxial region and consequent reduction in leaf angle. Silencing *NtPIN3* recapitulated the *NtTAC1* disruption phenotype, confirming that the *NtTAC1‐NtPIN3* axis regulates both auxin asymmetry and cell wall remodelling. Consistently, both knockdown lines exhibited enhanced lignin deposition, linking disrupted auxin flow to secondary wall thickening. Moreover, CRISPR/Cas9‐mediated editing of *SlTAC1* in tomato suppressed *SlPIN3* expression, indicating evolutionary conservation of this module. Collectively, our findings uncover a cell‐type‐resolved mechanism underlying leaf angle regulation and provide a mechanistic framework for precision engineering of crop architecture adapted to high‐density cultivation.

## Graphical abstract

Proposed model of *TAC1*‐mediated leaf angle regulation in tobacco.

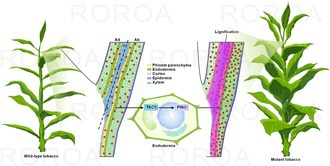

## 1 Introduction

Plant architecture, encompassing the three‐dimensional arrangement of vegetative and reproductive organs, is a foundational determinant of crop productivity and resilience in modern agriculture. Optimized architecture directly governs critical physiological processes such as light interception, photosynthetic efficiency, resource utilization, and canopy microclimate. This translates into tangible benefits including enhanced yield potential under high‐density planting, improved resource use efficiency (light, water, nutrients), and reduced susceptibility to pests and diseases through better airflow and light penetration, which is a cornerstone of sustainable green crop protection strategies (Hu et al. 2024; Liu, Zhang, et al. 2024; Saeed et al. 2025). Consequently, tailoring plant architecture has emerged as a pivotal breeding objective to meet the escalating demands of global food security amidst climate challenges and resource constraints.

Among the key architectural traits, leaf angle, defined as the spatial relationship between the leaf midrib and stem, is a critical agronomic trait that shapes plant architecture by modulating light interception, photosynthetic efficiency, and yield potential (Tian et al. 2024; Cao et al. 2022). For instance, a near‐isogenic maize line carrying the LIGULELESS2 (*lg2*) allele displayed a highly erect leaf architecture, characterized by a leaf angle close to 10°, and achieved an increase in grain yield compared to genotypes with more horizontal leaves (Mantilla‐Perez and Salas Fernandez 2017). Similarly, a separate study demonstrated that reducing leaf angle led to an improvement in maize yield, highlighting the positive correlation between reduced leaf inclination and enhanced productivity under dense planting conditions (Lauer et al. 2012). These findings underscore the value of optimizing leaf architecture to minimize mutual shading and enhance productivity in modern cropping systems.

Genetic studies have revealed that leaf angle is regulated by a complex interplay of hormonal cues and transcriptional networks. In rice and maize, mutations in the conserved *LAZY1* gene, a key regulator of gravitropic response, disrupt auxin asymmetry in pulvinus parenchyma cells, leading to exaggerated leaf angles and impaired canopy structure that negatively affects light interception and may reduce yield (Wang, Huang, et al. 2023; Li et al. 2007). The *OsWRKY72*‐*OsMAPK6*‐*OsBRI1* signalling axis further illustrates how brassinosteroids (BRs) fine‐tune leaf architecture by enhancing BR sensitivity (Wang et al. 2024), offering a framework for engineering high‐density‐tolerant crop varieties.

Key regulators such as *TAC1* and *LAZY1* function antagonistically to modulate leaf angle via auxin redistribution: *TAC1* promotes outward leaf spreading, while *LAZY1* reinforces upright leaf posture by maintaining *PIN3* polarity and auxin asymmetry (Hollender et al. 2020; Zhang et al. 2018). In monocots like maize, leaf angle is also determined by lignin deposition in ligular sclerenchyma cells (Chen et al. 2024; Li, Gao, et al. 2021). Hormonal crosstalk further adds complexity: BRs interact with auxin signalling pathways via factors such as *OsBZR1*, which activates BR‐responsive genes while suppressing auxin efflux transporters, forming a feedback loop (Xia et al. 2021; Bai et al. 2007). Under high BR conditions, LIC, a C3H zinc‐finger protein, represses *BZR1* activity, thus maintaining hormonal homeostasis (Zhang et al. 2012). Environmental inputs, including nitrogen and phosphorus availability, modulate auxin homeostasis through genes like *OsGH3*‐1, which conjugates excess IAA, leading to erect leaf phenotypes under nutrient stress (Luo et al. 2023; Zhang et al. 2009).

Auxin, a central morphogen, regulates leaf angle by controlling differential cell elongation across the adaxial‐abaxial axis (Wang et al. 2022; Huang et al. 2021). This process involves polar auxin transport, mediated by PIN family proteins (Tan et al. 2016; Deslauriers and Spalding 2021), and the activation of auxin‐responsive genes, which together drive the asymmetric growth required for leaf angle development (Li, Sun, et al. 2021). In soybean, the distribution of auxin through *GmPIN1* regulates petiole angle and contributes to compact plant architecture (Zhang et al. 2022). Similarly, in rice, *TAC4* and *LAZY1* modulate auxin distribution to control both tiller and leaf angle (Li, Sun, et al. 2021; Huang et al. 2021). While the role of auxin in leaf angle regulation is well‐established in monocots (Li, Sun, et al. 2021), how these mechanisms operate at the cellular level in dicots remains largely unexplored. Recent studies highlight the complexity of hormonal crosstalk, where BRs interact with auxin pathways to fine‐tune leaf architecture (Nolan et al. 2020). These insights emphasize the importance of understanding auxin's role in leaf angle modulation to improve crop architecture and yield potential.

Recent advances in single‐nucleus transcriptomics and spatial omics have revolutionized our understanding of plant development by providing unprecedented resolution into cell‐type‐specific gene expression and metabolic landscapes (de Biron and Farrona 2025; Giacomello 2021). Single‐nucleus RNA‐seq has elucidated key processes such as stomatal lineage differentiation and shoot apical meristem cycling in *Arabidopsis thaliana* (Lopez‐Anido et al. 2021; Zhang, Žukauskaitė, et al. 2021), while cross‐species comparisons have revealed conserved xylem differentiation programs (Tung et al. 2023). Spatial transcriptomics complements these efforts by retaining tissue context and enabling the mapping of gene expression in situ (Zhong et al. 2025). Furthermore, integrating spatial metabolomics allows for cellular‐resolution insights into hormonal and metabolic reprogramming (Wang et al. 2021; Zhang, Chen, and Wang 2021). For instance, mass spectrometry imaging coupled with single‐nucleus RNA‐seq in *Taxus mairei* uncovered spatially resolved expression patterns for paclitaxel and phenolic acid biosynthesis (Zhan et al. 2023). These multi‐omics strategies offer powerful tools to dissect plant developmental processes and responses to environmental stimuli.

While the *LAZY1*‐*TAC1* antagonism in leaf angle regulation is well established in monocots, our understanding of *TAC1* function in dicots remains limited, particularly in terms of its spatial activity, signalling partners, and contribution to cell‐type‐specific auxin dynamics. In this study, we investigate *NtTAC1* in tobacco, a representative dicot species. Our results reveal that *NtTAC1* functions specifically in the endodermis, where it coordinates auxin redistribution via *NtPIN3*, an auxin transporter. This interaction leads to asymmetric auxin redistribution and increased lignin deposition, ultimately reducing leaf angle. Unlike the thin‐walled targeting observed in monocots, the endodermis localization of the *NtTAC1*‐*NtPIN3* module reflects a dicot‐specific regulatory niche. By integrating single‐nucleus transcriptomics, spatial metabolomics, and genetic perturbation, we uncover a spatiotemporally resolved mechanism underlying leaf angle control. These findings not only provide mechanistic insights into *TAC1* function in dicots but also offer new targets for the rational design of high‐density‐adapted crop varieties.

## 2 Results

### 2.1 *NtTAC1* Coordinates Cellular Remodelling and Hormonal Crosstalk to Regulate Leaf Angle

**FIGURE 1:**
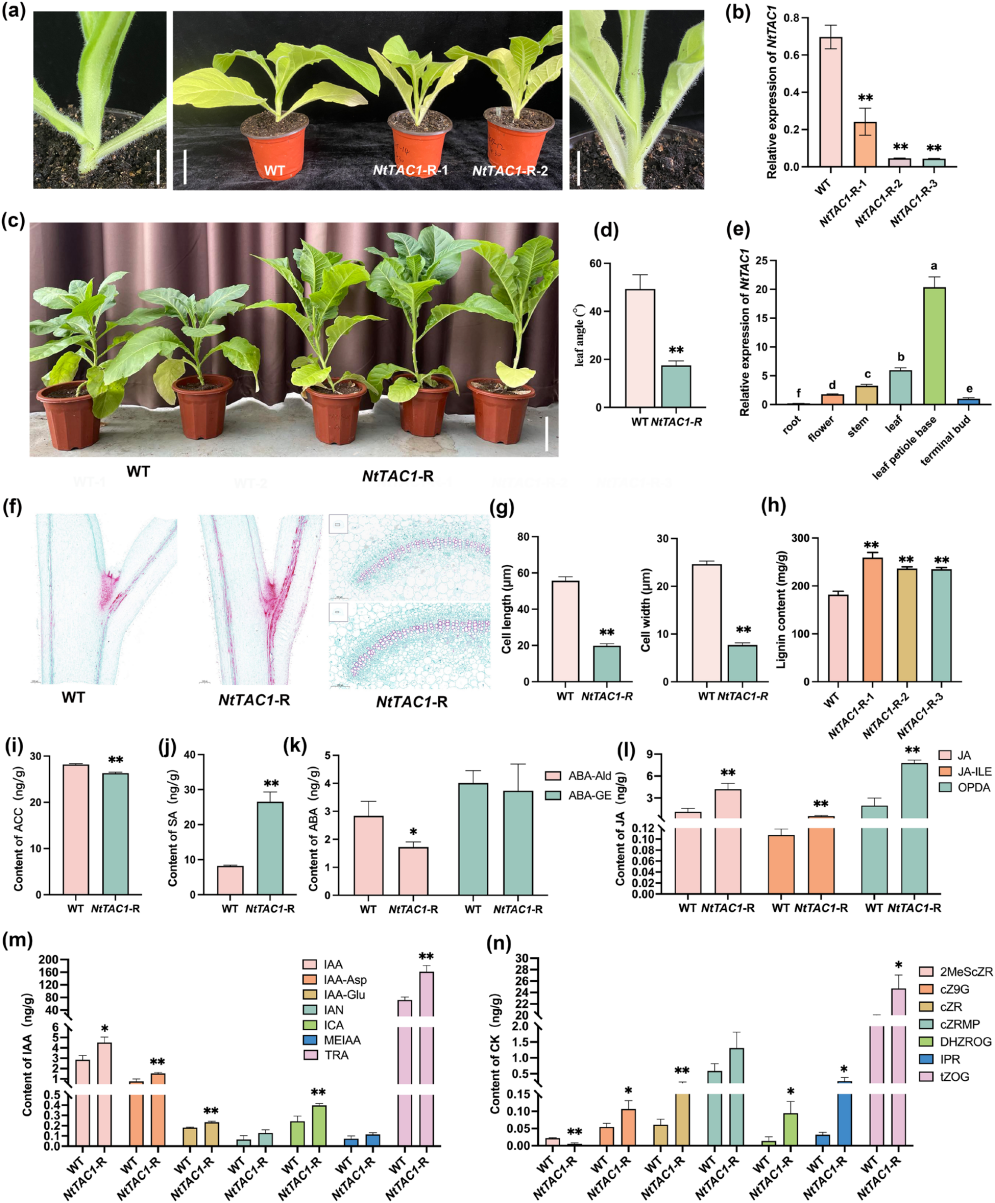
Suppression of *NtTAC1* alters leaf angle and cellular morphology in the pulvinus. (a) Leaf angle phenotypes of WT and *NtTAC1*‐R plants. Scale bar: 5 cm (center image), 15 cm (side images). (b) Relative expression levels of *NtTAC1* in WT and *NtTAC1‐*R plants. Data are presented as mean ± SD (*n* = 3). (c) Phenotypic comparison of WT and *NtTAC1‐*R plants. Scale bar = 10 cm. (d) Quantification of leaf angles in WT and *NtTAC1‐*R lines. Data are presented as mean ± SD (*n* = 12). (e) Relative expression levels of *NtTAC1* in various tissues of WT tobacco plants. Data are presented as mean ± SD (*n* = 3). (f) Cross‐sections of leaf angle in WT and *NtTAC1‐*R plants. Scale bar = 100 μm. (g) Quantification of cell width and length in the leaf angle of WT and *NtTAC1*‐R plants. Data are presented as mean ± SD (*n* = 3). (h) Lignin content in leaf angles of WT and *NtTAC1*‐R lines. Data are presented as mean ± SD (*n* = 3). (i–m) Hormonal analysis in the petiole base region of WT and *NtTAC1*‐R plants. Data are presented as mean ± SD (*n* = 3). Statistical significance was determined by Student's *t*‐test (b, d, g, h, i, k, l, m, n) and One‐way ANOVA followed by Tukey's test (e). **p < 0.01, *p < 0.05. *n* = biologically independent samples.

To elucidate the role of *NtTAC1* in leaf angle regulation, we generated stable *NtTAC1* RNAi (*NtTAC1*‐R) tobacco lines. *NtTAC1*‐R plants exhibited an average 63.7% reduction in leaf angle compared to wild‐type (WT) plants (Figure 1a–c), indicating that *NtTAC1* plays a critical role in regulating leaf architecture. Statistical analysis of leaf angle measurements is presented in Figure 1d. Tissue‐specific expression profiling showed that *NtTAC1* predominantly accumulates in the petiole base region (Figure 1e), which governs leaf angle formation. The subcellular localization of NtTAC1 in both the cytoplasm and nucleus is shown in Figure S1.

Histological examination of the lamina–petiole junction revealed pronounced cellular defects in *NtTAC1*‐R plants. Specifically, endodermal cells adjacent to vascular bundles displayed markedly reduced longitudinal length and radial width, suggesting impaired cell elongation and expansion (Figure 1f,g). Consistently, enhanced lignification was observed in *NtTAC1*‐R lines, with significantly elevated lignin accumulation in the petiole base compared to WT plants (Figure 1h). These results suggest that *NtTAC1* is required for coordinated cellular remodelling during leaf angle development.

To explore the hormonal basis for the altered leaf angle phenotypes in *NtTAC1*‐R plants, we quantified the levels of several key phytohormones (Figure 1i–n). Ethylene and abscisic acid levels were reduced, whereas salicylic acid, jasmonic acid, auxin, and cytokinin levels were elevated. Notably, the levels of indole‐3‐acetic acid (IAA) and its conjugates (IAA‐Asp and IAA‐Glu), as well as trans‐zeatin riboside (tZR), indole‐3‐carboxylic acid (ICA), and tryptamine (TRA), were significantly altered (Figure 1m). These findings indicate that *NtTAC1* modulates leaf angle through coordinated regulation of hormone balance and crosstalk.

### 2.2 Single‐Nucleus Transcriptomics Resolves Cell‐Type Composition of Leaf Angle Tissue

**FIGURE 2:**
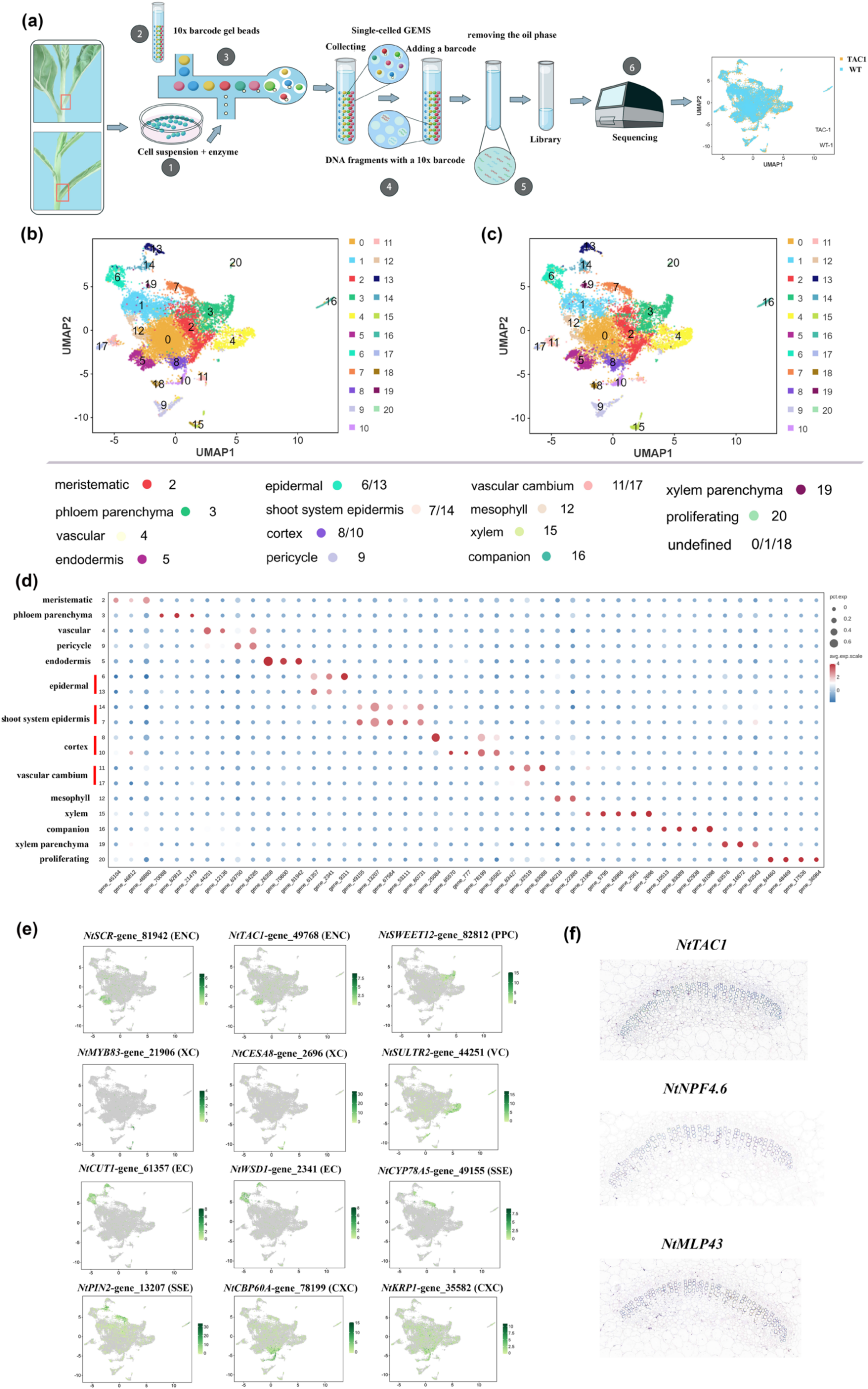
SnRNA‐seq profiles the cellular landscape of petiole base region formation in WT and *NtTAC1*‐R plants. (a) Overview of the tobacco petiole base region snRNA‐seq workflow. Nuclei were isolated from the petiole base region of WT and *NtTAC1*‐R plants. (b, c) UMAP plots illustrating the distribution of cells of tobacco petiole base region in WT (b) and *NtTAC1*‐R (c) plants, including meristematic cells (SMC, cluster 2); phloem parenchyma cells (PPC, cluster 3); vascular cells (VC, cluster 4); endodermal cells (ENC, cluster 5); epidermal cells (EC, clusters 6 and 13); shoot system epidermal cells (SSE, clusters 7 and 14); cortex cells (CXC, clusters 8 and 10); pericycle cells (PEC, cluster 9); vascular cambium cells (VCC, clusters 11 and 17); mesophyll cells (MC, cluster 12); xylem cells (XC, cluster 15); companion cells (CC, cluster 16); xylem parenchyma cells (XP, cluster 19); and proliferating cells (PC, cluster 20). (d) Dot plots illustrating the expression of key marker genes across different cell types. (e) UMAP plots showing the expression patterns of specific genes across different cell types in WT and *NtTAC1*‐R plants. (f) RNA in situ hybridization of expression patterns of key genes (*NtTAC1*, *NtNPF4.6*, and *NtMLP43*) in specific cell types. Scale bars = 200 μm.

Leaf angle tissue is structurally complex and highly lignified, which limits efficient protoplast isolation. To resolve its cellular composition and regulatory landscape, we performed single‐nucleus RNA sequencing (snRNA‐seq) on the petiole base region of WT and *NtTAC1*‐R plants (Figure 2a). Two single nuclear libraries were constructed using the 10× Genomics Chromium platform. A total of 813 361 258 clean reads were obtained from transcriptomes of 26 865 cells, achieving an average read count of > 30 275 per cell. The median number of genes per cell nucleus was > 2300, with a median unique molecular identifier (UMI) count > 2800. The high‐quality read ratio at Q30 exceeded 95.1%. In total, 11 985 individual cells were profiled for WT and 14 880 individual cells for *NtTAC1*‐R groups (Table S1). The average gene number and UMI in petiole base region cells identified were shown in Figure S2.

After quality filtering and integration of both genotypes, 27 735 genes were retained for downstream analysis. Graph‐based clustering identified 20 transcriptionally distinct clusters (Figure 2b,c). Cluster correlation and cellular composition analysis were shown in Figure S3. Dimensionality reduction using UMAP and t‐SNE revealed clear separation among clusters. Based on known marker genes and cluster‐specific expression patterns, these clusters were annotated into 14 major cell types within petiole base region tissue (Figure S4). To differentiate various cellular populations, we focused on identifying genes that exhibit a significant increase in expression within specific cell clusters, while their expression levels are comparatively lower in other cell clusters. Within these clusters (*n* = 14), we selected 47 distinctive marker genes, including those associated with endodermis development (Figure 2d and Table S2). To validate our cluster annotations experimentally, we performed RNA in situ hybridization targeting key marker genes such as *NtTAC1*, *NtNPF4.6*, and *NtMLP43*, confirming their endodermis‐specific expression (Figure 2e,f).

### 2.3 The Endodermis as a Central Hub Mediating *NtTAC1* Regulation of Leaf Angle

**FIGURE 3:**
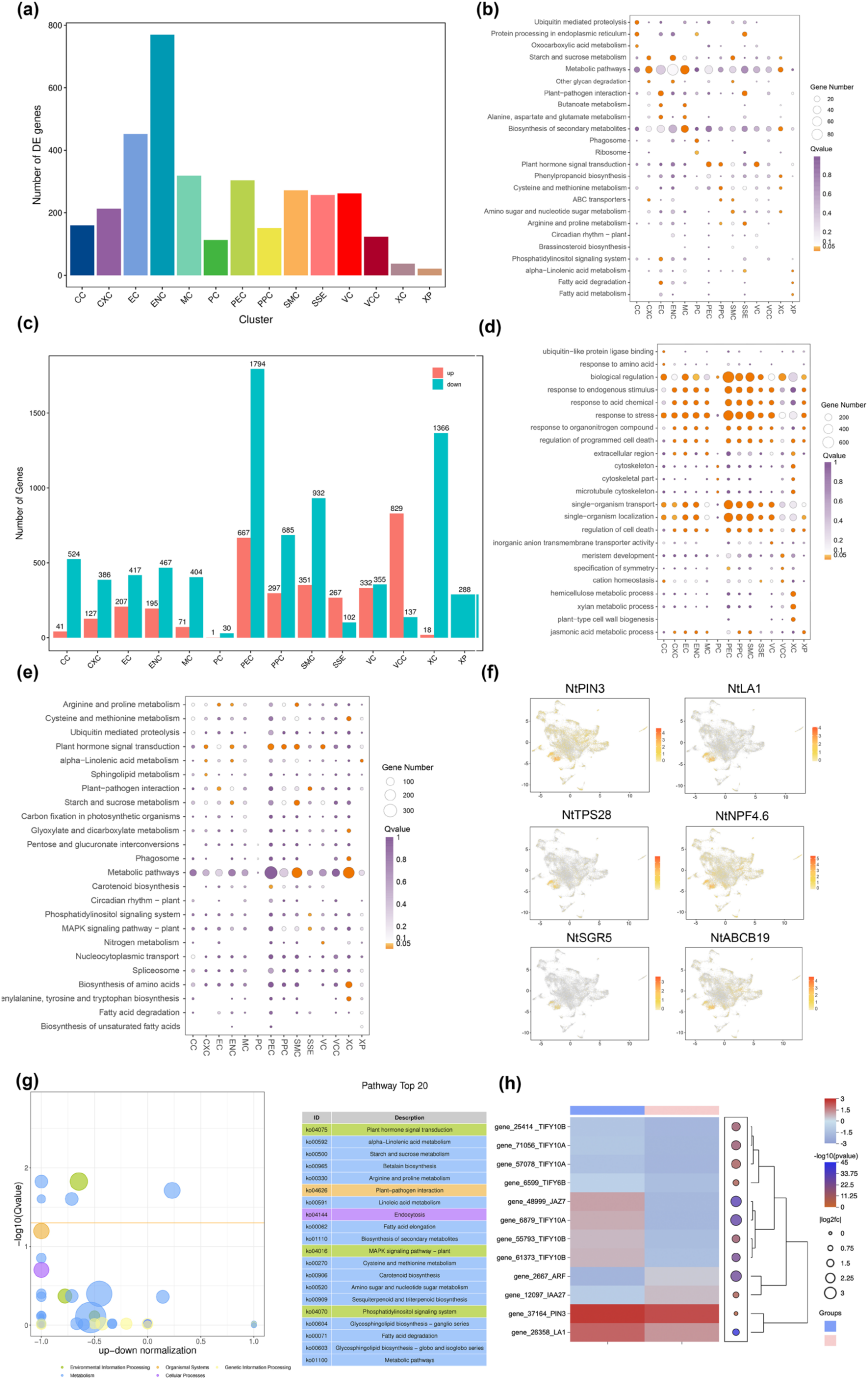
Cell‐type‐specific response to *NtTAC1* suppression in the tobacco leaf angle. (a) Bar chart showing the number of up‐regulated genes in each identified cell subpopulation. (b) KEGG pathway enrichment analysis of cell‐type‐specific marker genes. (c) Bar chart illustrating the number of DEGs between WT and *NtTAC1*‐R plants in each cell type. (d) GO enrichment analysis of DEGs in different cell types. (e) KEGG pathway enrichment analysis of DEGs in different cell types. (f) UMAP heatmap displaying the expression patterns of key maker genes in endodermis (cluster 5). (g) KEGG pathway enrichment analysis of DEGs in the endodermis. (h) Heatmap showing the expression levels of hormone‐ and leaf angle‐related regulatory genes in WT and *NtTAC1*‐R plants across different cell types.

To investigate the cell‐type‐specific effects to *NtTAC1* in tobacco leaf angle regulation, we integrated single‐nucleus transcriptomic profiles from WT and *NtTAC1‐R* plants (Figure 3a). To explore the molecular signatures of specific cell subpopulations, we focused on genes that were significantly upregulated within these subpopulations (Figure 3b). Cell‐type‐specific KEGG pathway enrichment identified relevant biological pathways, such as “starch and sucrose metabolism”, “other glycan degradation”, and “plant hormone signal transduction” in the endodermis, reflecting its role in energy metabolism and hormonal regulation. Xylem markers were enriched in pathways associated with “amino sugar and nucleotide sugar metabolism” and “phenylpropanoid biosynthesis”, driving secondary cell wall formation, while meristem markers highlighted “ABC transporters”, supporting active nutrient partitioning (Figure 3b and Table S3).

To identify whether the expression levels of certain genes are influenced by *NtTAC1* in a manner that is specific to different cell types, we identified 53 408 differentially expressed genes (DEGs) across cell types between *NtTAC1*‐R and WT samples (Figure 3c and Table S4). Notably, pericycle (2461 DEGs), xylem (1384 DEGs), and meristematic cells (1283 DEGs) showed the highest sensitivity (Figure 3c). Gene Ontology (GO) analysis revealed that the jasmonic acid metabolic pathway was enriched in multiple cells, indicating that the leaf angle was synergistically affected by multiple hormones (Figure 3d and Table S5). KEGG analysis further demonstrated broad activation of “plant hormone signal transduction” across six cell types, while “starch and sucrose metabolism” was upregulated in endodermis and meristem, likely supporting cell wall biosynthesis (Figure 3e and Table S6).

Among all the cell subpopulations, the endodermis has the highest number of upregulated genes (Figure 3a), suggesting the activation of a prominent regulatory pathway in this cell type. Endodermis markers, including *NtSCR*, *NtTAC1*, *NtLAZY1* (*NtLA1*), and *NtPIN3*, showed distinct, cell‐type‐specific expression patterns (Figure 3f). *NtTAC1* and *NtLA1* are known regulators of leaf angle, while *NtPIN3* emerged as a novel auxin transporter localized to this layer. This co‐localization underscores the endodermis as a central regulatory hub for leaf angle determination, with *NtPIN3*‐mediated auxin transport playing a pivotal role in coordinating cellular remodelling and hormonal crosstalk. KEGG enrichment analysis of differential genes in the endodermis further revealed significant enrichment in plant hormone signal transduction (Figure 3g and Table S7). In *NtTAC1*‐R plants, expression of auxin‐related genes was higher, while *NtLA1* expression decreased, consistent with changes in *NtPIN3* expression (Figure 3h).

### 2.4 *NtTAC1* Regulates Leaf Angle Formation Through Cell‐Type‐Specific Auxin Signalling

**FIGURE 4:**
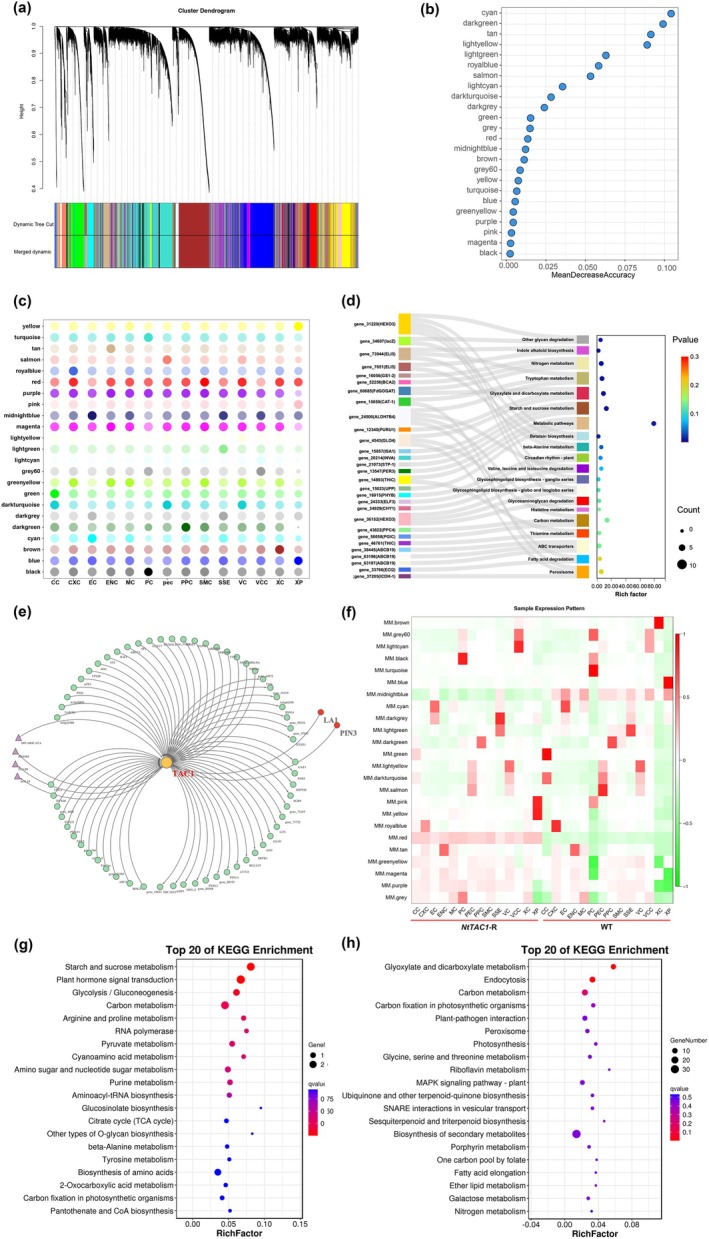
Cell‐type‐specific regulation of leaf angle by *NtTAC1*. (a) Hierarchical clustering of co‐expressed gene modules identified by hgWGCNA. Coloured bars indicate module assignments. (b) Accuracy of cell type classification was assessed using Random Forest modelling. (c) Cell‐type‐specific expression patterns across distinct gene modules. (d, e) Functional characterization of the tan module: (d) KEGG enrichment of genes in the tan module. (e) Subnetwork visualization highlighting the hub gene *NtTAC1* and its co‐expressed partners. (f) Inter‐module correlation heatmap illustrating synergistic and antagonistic relationships between gene modules. (g) KEGG enrichment analysis of genes in red module. (h) KEGG enrichment analysis of genes in midnightblue module.

To delineate the cell‐type‐specific regulatory roles of *NtTAC1* in transcriptional networks, we performed high‐dimensional weighted gene co‐expression network analysis (hdWGCNA) at single‐nucleus resolution. Topological analysis partitioned the co‐expression network into 25 modules, with sizes ranging from 90 to 3129 co‐regulated transcripts (Figure 4a and Table S8). Random Forest‐based machine learning framework identified four co‐expression modules (cyan, darkgreen, tan, lightyellow) with maximal discriminative capacity between WT and *NtTAC1*‐R leaf angle phenotypes (Figure 4b). The cyan, darkgreen, and tan modules exhibited cell‐type‐specific enrichment, displaying dominant expression signatures in epidermal, phloem parenchyma, and endodermal cells, respectively (Figure 4c and Table S9).

KEGG pathway enrichment analysis revealed the tan module was significantly enriched in auxin‐related pathways, including tryptophan metabolism, nitrogen metabolism, ABC transporters, with concurrent enrichment of the biosynthesis of indole alkaloids (Figure 4d). Notably, this module contained *NtPIN3*, a pivotal auxin efflux carrier gene, highlighting its central role in modulating auxin flux dynamics during leaf angle development. Analysis of the *NtTAC1* co‐expression network, based on hub genes from the tan module, identified significant associations with four transcription factors: *ERF003*, *TULP8*, *AGL19*, and *DIVARICATA*. This network analysis demonstrated that *NtTAC1* exhibits strong co‐expression relationships with key regulatory genes, including *NtLA1* and *NtPIN3*, with *NtLA1* being a well‐characterized determinant of leaf angle regulation in plants (Figure 4e).

To further explore these findings, we generated a heat map of sample expression patterns (Figure 4f). The red module was consistently upregulated in *NtTAC1*‐R plants across all cell types, with functional enrichment in starch/sucrose metabolism and plant hormone signalling, including several key auxin biosynthesis genes. This suggests *NtTAC1* may regulate leaf angle through the modulation of growth‐related hormone pathways (Figure 4g and Table S10). Conversely, the midnight module was globally downregulated in *NtTAC1‐*R plants, with significant suppression of carbon metabolism, carbon fixation in photosynthetic organisms, and photosynthesis (Figure 4h and Table S11).

### 2.5 Developmental Trajectory Analysis Identifies *NtPIN3* as a Key Regulator in *NtTAC1* ‐Mediated Endodermis Differentiation

**FIGURE 5:**
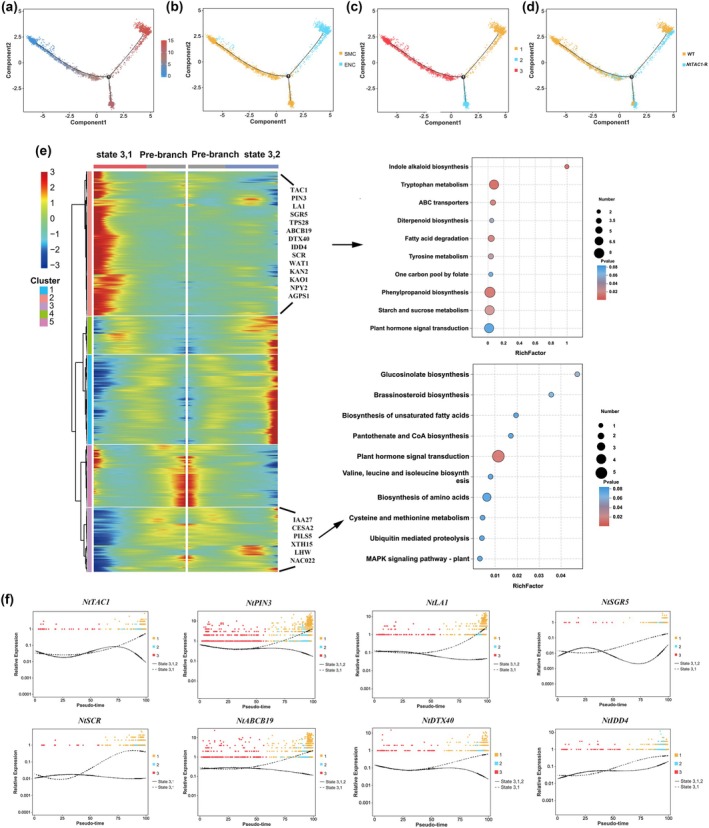
Molecular and cellular differentiation analysis of WT and *NtTAC1*‐R plants at the single‐nucleus level. (a) Pseudotime trajectory of cell differentiation. Single cells are arranged along the developmental continuum from the meristematic tissue to the endodermis. The colour gradient represents pseudo‐time, and smaller values indicate earlier developmental stages. (b) Cell subpopulation analysis of cell differentiation relationship. Colours represent cell subpopulations identified through clustering analysis. (c) State trajectory of cell differentiation. The colour represents different differentiation states during the differentiation process. (d) Sample distribution of cell differentiation states. The colour represents the biological sample (blue: WT; red: *NtTAC1*‐R). (e) Dynamic gene expression heat map and KEGG pathway enrichment analysis. Left image: Heat map of DEGs during meristem‐to‐endodermis differentiation. Row represent genes (clustered by expression pattern), column represent cells sorted by pseudotime. Expression *z*‐score display (blue: Low; red: Tall). Right: KEGG pathway enrichment analysis of DEGs along pseudotime. (f) Trajectory plot of expression levels of DEGs along pseudotime. The expression trends of representative DEGs are grouped by functional category. The shape of the lines represents different branches.

Leaf angle formation begins in the meristematic region, where dynamic cell division and differentiation shape leaf orientation. Although *NtTAC1* is known to influence leaf angle, its role in early cell fate decisions remains unclear. To address this, we performed single‐nucleus transcriptome analysis focusing on meristematic and endodermal cells. Based on marker gene expression, clusters 2 and 5 were identified as the meristem and endodermis, respectively. Pseudotime trajectory analysis revealed a clear progression from meristematic to endodermal cells, indicating a directional differentiation path (Figure 5a,b). This trajectory included one branch point and comprised three developmental states (Figure 5c). Comparison of genotypes revealed that WT cells were primarily located in early pseudotime states, while *NtTAC1*‐R cells were enriched in later states associated with endodermal identity, suggesting that *NtTAC1* suppression promotes premature differentiation of endodermal cells (Figure 5d).

To further identify regulators of this transition, we analysed differentially expressed genes along the pseudotime trajectory, grouped them into five clusters based on their expression patterns (Figure 5e). Cluster 2 contained genes whose expression increased during meristem‐to‐endodermis differentiation, including *NtTAC1*, *NtLA1*, *NtABCB19*, and the auxin transporter *NtPIN3*, all of which have been implicated in leaf angle regulation. KEGG enrichment analysis of cluster 2 genes revealed a significant overrepresentation in tryptophan metabolism, indole alkaloid biosynthesis, and plant hormone signal transduction, highlighting the central role of auxin regulation in endodermis fate specification. Scatter plots of gene expression along pseudotime showed that *NtPIN3* and the other four genes were upregulated during this transition in WT plants, but remained low in RNAi lines (Figure 5f). These results suggest that *NtTAC1* is required for proper activation of these genes during endodermal cell fate specification.

### 2.6 *NtTAC1* Disrupts Auxin Polarization via *NtPIN3* to Drive Leaf Angle Reduction

**FIGURE 6:**
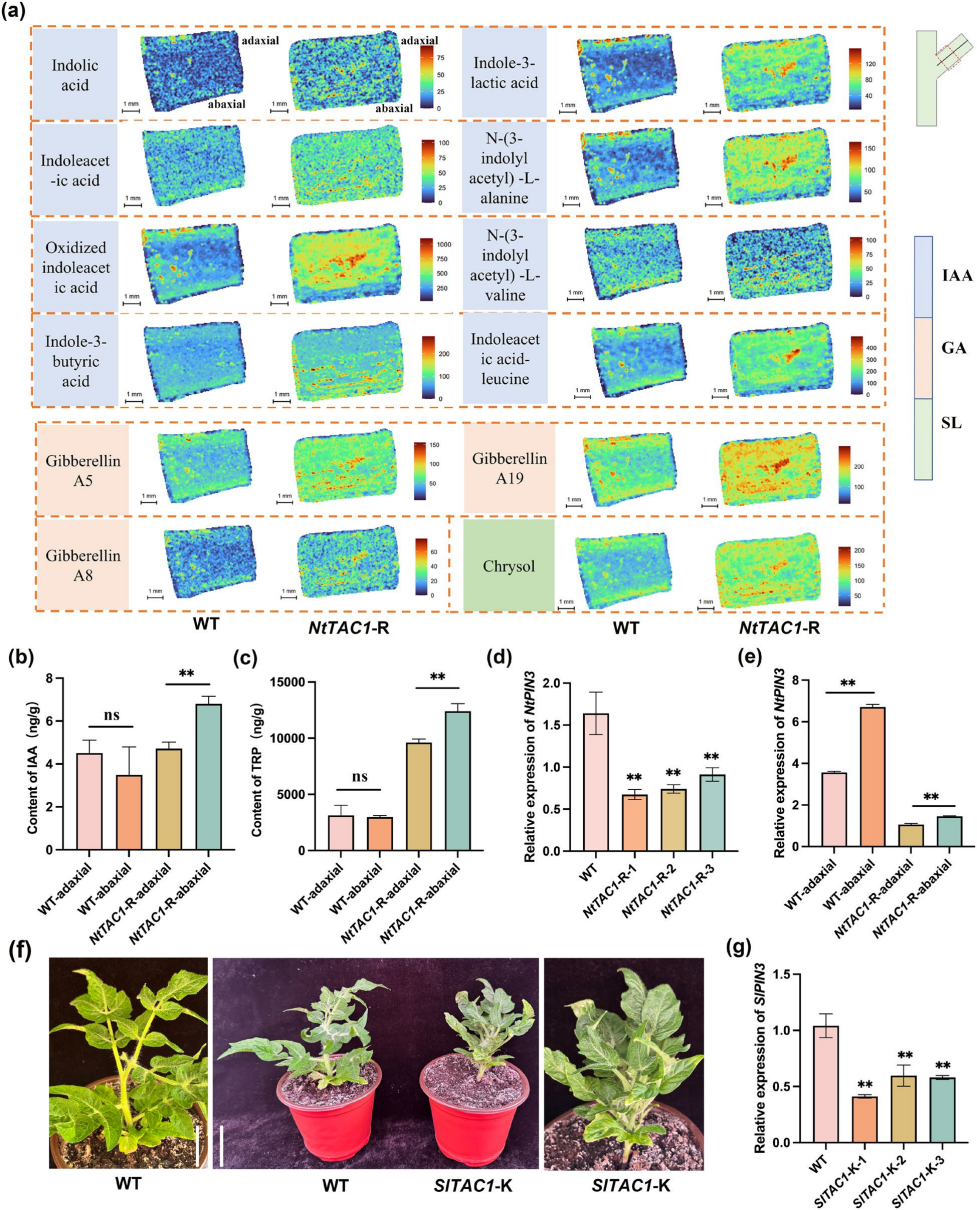
A conserved *TAC1*‐*PIN3* module directs auxin asymmetry to regulate leaf angle across species. (a) Spatial metabolic analysis of auxin (IAA), strigolactones (SLs), and gibberellins (GAs) in WT and *NtTAC1*‐R lines. (b, c) Quantification of IAA and TRP content in the adaxial and abaxial regions of leaf angle tissues of WT and *NtTAC1‐*R lines. Data are presented as mean ± SD (*n* = 3). (d) Relative expression levels of *NtPIN3* in the leaf angle of WT and *NtTAC1‐*R lines. Data are presented as mean ± SD (*n* = 3). (e) Relative expression levels of *NtPIN3* in the adaxial and abaxial regions of leaf angle tissues of WT and *NtTAC1*‐R lines. Data are presented as mean ± SD (*n* = 3). (f) Leaf angle phenotypes in WT and *SlTAC1*‐K plants. Scale bar: 5 cm (center image), 15 cm (side images). (g) Relative expression levels of *SlTAC1* in the leaf angle of WT and *SlTAC1*‐K lines. Data are presented as mean ± SD (*n* = 3). Statistical significance was determined by Student's *t*‐test. **p < 0.01. *n* = biologically independent samples.

Spatial metabolomics revealed pronounced alterations in hormone distribution patterns within the petiole base region of *NtTAC1*‐R plants relative to WT. Notably, IAA and its derivatives (oxIAA, IBA) displayed asymmetric accumulation, with significantly higher levels in the abaxial region (Figure 6a). This abaxial‐biased auxin accumulation indicates that *NtTAC1* is required to maintain proper auxin polarity during leaf angle formation. To investigate the metabolic basis of this auxin redistribution, we quantified TRP and IAA levels in the adaxial and abaxial regions of the petiole base region. *NtTAC1*‐R plants exhibited a clear abaxial‐biased enrichment of both metabolites, with significantly higher levels in the abaxial side (Figure 6b,c). These data suggest that *NtTAC1* influences local auxin biosynthesis and/or distribution dynamics, contributing to spatial auxin asymmetry. In addition to auxin, gibberellins (GAs) and strigolactones (SLs) were increased levels in *NtTAC1*‐R plants, with significant adaxial–abaxial differences (Figure 6a), indicating broader hormonal reprogramming associated with *NtTAC1* perturbation.

This altered auxin distribution was accompanied by a significant reduction in *NtPIN3* expression in *NtTAC1*‐R plants (Figure 6d), suggesting that *NtTAC1* contributes to the maintenance of auxin polarity through regulation of *NtPIN3* transcription. To further investigate this mechanism, we examined *NtPIN3* expression on the abaxial and adaxial sides of the petiole base region. In WT plants, *NtPIN3* exhibited a clear polar distribution, with significantly higher expression on the abaxial side than on the adaxial side. In *NtTAC1*‐R plants, although abaxial expression remained significantly higher than adaxial expression, the levels on both sides were substantially lower than those on the corresponding sides in WT plants, resulting in a significantly reduced abaxial/adaxial expression ratio (Figure 6e). These results indicate that *NtTAC1* is required for both maintaining *NtPIN3* transcript levels and establishing its proper polar distribution.

To evaluate the conservation of this regulatory mechanism, we generated *SlTAC1*‐edited (*SlTAC1*‐K) tomato lines using CRISPR/Cas9. These lines exhibited reduced leaf angle (Figure 6f) and decreased expression of *SlPIN3* (Figure 6g). The mutation types of *SlTAC1* are shown in Figure S5. Tissue‐specific expression analysis revealed that *SlTAC1* is expressed in various tissues, with the highest transcript level detected in the leaf angle region (Figure S6), consistent with its role in leaf angle regulation.

### 2.7 *NtPIN3* Silencing Reduces Leaf Angle by Altering Auxin Distribution and Lignin Accumulation

**FIGURE 7:**
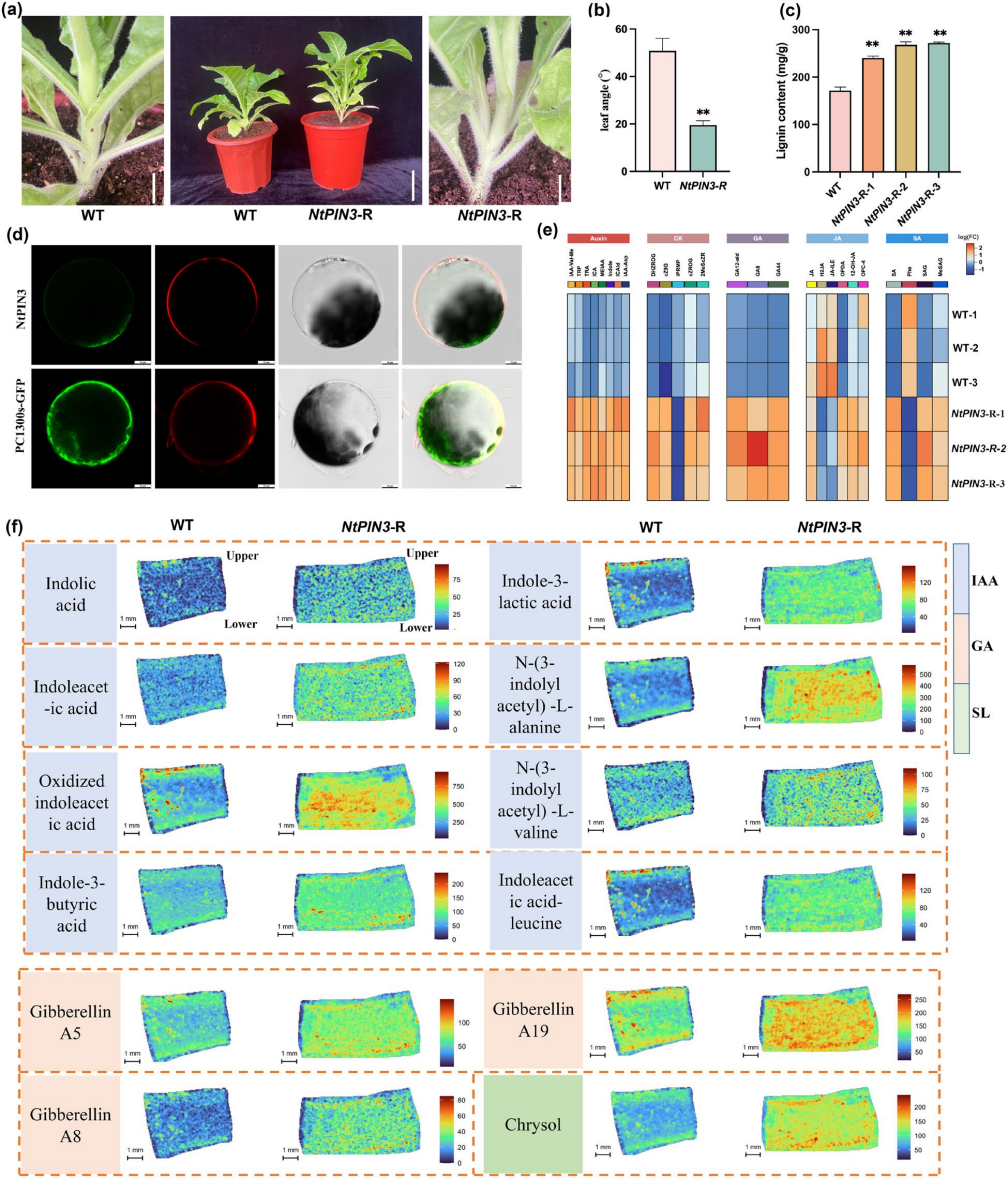
Characterization of *NtPIN3* function in leaf angle development. (a) Phenotypic comparison of leaf angles between WT and *NtPIN3‐R* plants. Scale bar: 4 cm (center image), 16 cm (side images). (b) Quantification of leaf angles in WT and *NtPIN3‐R* plants. Data are presented as mean ± SD (*n* = 12). (c) Lignin content in leaf angles of WT and *NtPIN3‐R* lines. Data are presented as mean ± SD (*n* = 3). (d) Subcellular localization of NtPIN3‐GFP fusion protein in endodermal cells. (e) Spatial metabolic analysis of auxin distribution in WT and *NtPIN3‐R* lines. (f) Hormonal analysis in WT and *NtPIN3‐R* plants. Statistical significance was determined by Student's *t*‐test. **p < 0.01. *n* = biologically independent samples.

To investigate the role of *NtPIN3* in leaf angle development, we first examined its tissue‐specific expression pattern and found that *NtPIN3* transcripts accumulated in large quantities in leaf petiole base (Figure S7). In addition, we utilized RNAi technology to generate *NtPIN3* interference transgenic lines (*NtPIN3*‐R), as phylogenetic tree analysis identified three *NtPIN3* genes with > 80% homology, and expression levels of all three genes were significantly reduced in these lines, resulting in a decrease of total *NtPIN3* transcripts (Figure S8). *NtPIN3‐R* lines exhibited a significant reduction in leaf angle compared to WT plants (Figure 7a,b). This phenotype closely resembled that observed in *NtTAC1*‐R plants. Subcellular localization assays indicated that NtPIN3 is primarily localized at the plasma membrane (Figure 7d), consistent with its predicted role in auxin transport.

Additionally, we observed increased lignin accumulation in the leaf corners of *NtPIN3‐R* plants, as confirmed by biochemical analysis (Figure 7c), consistent with the lignin changes observed in *NtTAC1*‐R plants. Heatmap analysis of hormone content revealed a significant increase in auxin levels in *NtPIN3*‐R lines compared to WT, highlighting the impact of *NtPIN3* silencing on auxin distribution (Figure 7e). Spatial metabolic analysis further showed elevated accumulation in *NtPIN3*‐R plants, with asymmetric distribution along the adaxial–abaxial axis, where the abaxial region exhibited significantly higher auxin levels than the adaxial region (Figure 7f). This distribution pattern was similar to that observed in *NtTAC1*‐R plants. Moreover, the changes in GAs and SLs hormone levels were also consistent with those observed in *NtPIN3*‐R plants (Figure 7f), suggesting that the altered hormonal distribution contributes to the reduced leaf angle phenotype of *NtPIN3*‐R plants.

## 3 Discussion

### 3.1 Evolutionary Divergence in Leaf Angle Regulation Mechanisms

Leaf angle, defined as the spatial relationship between the stem and leaf midrib, is a critical agronomic trait determining crop yield potential through optimized light interception and canopy architecture (Mantilla‐Perez and Salas Fernandez 2017). Monocot studies (e.g., maize *ZmTAC1* [Ku et al. 2011] and rice *OsTAC1* [Yu et al. 2007]) have established conserved mechanisms of leaf angle regulation via auxin redistribution and cell division control in pulvinus tissues. Although initially characterized in monocots, *TAC1* has also been implicated in regulating shoot architecture in a variety of dicots. For instance, in peach ( *Prunus persica* ), mutations in *PpeTAC1* result in upright growth habits due to reduced branch angles (Dardick et al. 2013). Similarly, in *Arabidopsis*, loss‐of‐function mutations in *AtTAC1* lead to narrower branch angles, indicating a conserved role of *TAC1* in promoting wider branch orientations across diverse plant species (Hollender et al. 2020). Importantly, findings in *Arabidopsis* also highlight a distinct cellular niche for shoot gravitropism regulation: the endodermis, which serves as a critical site for gravity sensing and auxin redistribution (Fukaki et al. 1998).

In our study, we identify *NtTAC1* as a novel regulator of leaf angle in tobacco ( *Nicotiana tabacum* ). Unlike monocot *TAC1* genes that primarily function in pulvinus parenchyma cells, *NtTAC1* predominantly operates in endodermal cells, modulating auxin signalling and cell wall properties. This tissue‐specific activity underscores an evolutionary divergence in the regulatory mechanisms of leaf angle between monocots and dicots.

### 3.2 Endodermis as the Epicentre of *NtTAC1* Action

The endodermis has recently emerged as a signalling hub integrating hormonal and mechanical cues (Doblas et al. 2017; Geldner 2013). Here, we uncover an unexpected role for this cell layer, that *NtTAC1* acts specifically in the endodermis to regulate leaf angle in tobacco. Several lines of evidence support this cell‐type‐specific mechanism. First, co‐expression network analysis identified *NtTAC1* and the auxin transporter *NtPIN3* as co‐regulated hubs within an endodermis module (Figure 4), suggesting that *NtTAC1* modulates local auxin gradients directly within this tissue. Second, pseudotime trajectory analysis revealed that both genes promote endodermal cell differentiation, with *NtPIN3* acting as a key checkpoint (Figure 5). The marked increase in endodermal cell number in *NtTAC1*‐R plants further confirms that *NtTAC1* exerts direct control over endodermal fate determination.

The preference for the endodermis can be attributed to its unique physiological features. This cell layer is intrinsically equipped to perceive gravitational signals, a key input for leaf angle control, and simultaneously serves as a central hub for auxin transport (Dinneny 2014). *NtTAC1* appears to exploit both properties: it integrates mechanical sensing with local auxin modulation via *NtPIN3*, thereby coordinating differential cell expansion at the lamina joint. The cell‐autonomous expression of *NtTAC1*, *NtPIN3*, and *NtLA1* within endodermal cells further supports this model.

This cell‐autonomous mechanism in the endodermis contrasts with non‐cell‐autonomous signalling pathways, such as the “auxin‐*PLT5*‐*SND1*” axis regulating wood formation in poplar (Liu et al. 2025), and also differs from previously reported leaf angle regulators in monocots that act in parenchyma or vascular tissues (Teng et al. 2023; Liu, Li, et al. 2024). Our findings establish the endodermis as a novel regulatory center for leaf architecture in dicots and reveal how a single cell type integrates mechanical perception, hormonal signalling, and differentiation to shape organ geometry.

### 3.3 *NtTAC1* Disruption Alters Auxin Levels and Lignin Deposition in Leaf Angle Regulation

Auxin is a well‐established key player in regulating leaf angle by influencing cell elongation, division, and the positioning of leaf primordia (Liu et al. 2019; Mroue et al. 2018; Zheng et al. 2023). In our study, we observed that the tobacco *NtTAC1*‐R plants exhibited an increased auxin level and a reduced leaf angle, which was consistent with findings from other species, such as *Arabidopsis* and rice, where altered auxin homeostasis similarly affected leaf architecture (Kim et al. 2023; Ji et al. 2022). This reduction in leaf angle correlates with the disruption of *NtTAC1*, suggesting that *NtTAC1* influences auxin biosynthesis and/or distribution dynamics.

Our single‐nucleus transcriptomic analysis further supported the role of auxin in leaf angle regulation. KEGG enrichment analysis of differentially expressed genes (DEGs) between *NtTAC1*‐R and WT plants revealed significant enrichment in plant hormone signalling pathways, particularly those related to auxin, confirming that auxin distribution is disturbed in the absence of *NtTAC1*. Specifically, we observed upregulation of auxin biosynthesis genes, suggesting that *NtTAC1* disruption results in increased auxin production, particularly on the abaxial side, which would contribute to the observed leaf angle phenotype. These findings are in line with previous studies that demonstrate how altered auxin production influences leaf architecture (Gao et al. 2021; Zhang et al. 2018).

In addition to changes in auxin levels, we also observed a significant increase in lignin deposition in the *NtTAC1*‐R plants. This observation is consistent with the work of Sun et al. (2020) and Liu, Li, et al. (2024), who reported that high auxin levels can trigger increased lignin biosynthesis. This increase in lignin likely contributes to the mechanical reinforcement of the leaf tissues, further influencing leaf angle by restricting cell expansion. This observation also aligns with findings from Wang, Li, et al. (2023), who showed that altered auxin distribution can induce changes in cell wall properties, including lignin deposition, leading to changes in leaf morphology.

### 3.4 Asymmetric Auxin Distribution and Its Role in Leaf Angle Regulation

The spatial distribution of auxin within the leaf is a fundamental determinant of both leaf polarity and the angle of leaf emergence (Burian et al. 2022). Our study revealed a clear auxin asymmetry in *NtTAC1*‐R leaves, with higher auxin levels on the abaxial side compared to the adaxial side (Figure 6A). This pattern was confirmed by spatial metabolomics analysis, further supporting that *NtTAC1* disruption alters auxin dynamics within the leaf (Figure 6B). In our study, we observed significant downregulation of *NtPIN3* expression in the leaf angle region of *NtTAC1*‐R plants. Moreover, interference with *NtPIN3* led to a further reduction and an asymmetric distribution of auxin in the leaf angle, highlighting its critical role in shaping leaf architecture. This result aligns with previous studies showing that *PIN*‐mediated polar auxin distribution in species like *Arabidopsis* and rice is crucial for regulating plant growth responses, such as phototropism and gravitropism (Han et al. 2021; Gao et al. 2021; Kleine‐Vehn et al. 2010; Friml et al. 2002). More importantly, consistent downregulation of *SlPIN3* coupled with reduced leaf angles in *SlTAC1*‐K tomato plants supports a conserved role for *TAC1* in regulating *PIN3*‐mediated auxin redistribution in the dicot species examined here. However, the precise molecular mechanism by which *NtTAC1* orchestrates *NtPIN3* expression, whether through direct transcriptional regulation, epigenetic modification, or intermediary signalling cascades, remains to be deciphered. Future studies employing chromatin immunoprecipitation sequencing (ChIP‐seq) and promoter bashing assays will elucidate this regulatory hierarchy.

## 4 Conclusion

In summary, our study uncovers a dicot leaf angle regulatory framework centered on *NtTAC1*, which coordinates endodermal cell elongation, auxin asymmetry, and lignin deposition, representing a distinct strategy relative to monocot pulvinus‐based mechanisms. Developmental trajectory and WGCNA analyses identify *NtPIN3* as a key effector, with phenotypic convergence observed in both *NtTAC1*‐R and *NtPIN3*‐R plants, supporting a hierarchical genetic relationship between them. This module reflects evolutionary divergence in auxin‐mediated morphogenesis, whereby cell‐type‐specific regulation replaces ancestral tissue‐level control. The *NtTAC1*‐*NtPIN3* axis provides a potential molecular entry point for optimizing canopy architecture while maintaining mechanical resilience through lignin modulation.

## 5 Materials and Methods

### 5.1 Plant Materials and Growth Conditions

Seeds of WT and *NtTAC1*‐silenced (*NtTAC1*‐R) T1 transgenic plants were sown in 15 cm‐diameter pots containing a uniform substrate mixture (nutrient soil: vermiculite = 3:1, 2.5 kg per pot). All plants were grown in a greenhouse under controlled environmental conditions: temperature maintained at 22°C–25°C, 16‐h light/8‐h dark photoperiod with supplemental LED lighting (150 μmol m^−2^ s^−1^), and relative humidity at 60%–70%. Watering was applied consistently to maintain substrate moisture.

Gene expression pattern analysis was conducted using materials from 4‐month‐old WT plants, including roots, stems, leaves, flowers, apical buds, and leaf angle regions. Total RNA was extracted from each tissue type and used to synthesize cDNA for subsequent analysis.

### 5.2 Determination of Leaf Angle

Leaf angle was measured manually using a standard protractor under natural growth conditions. For each of the 12 tobacco lines, the fifth fully expanded leaf from the top of the main stem was selected at the fully expanded stage (10‐week‐old tobacco plants). With the stem as the reference axis, the measurement segment was selected from the natural bending point at the leaf axil to the leaf tip. Each leaf was measured three times with the average value calculated. Digital verification was simultaneously performed using ImageJ software on side‐view photographs to ensure measurement accuracy.

### 5.3 RNA Extraction and Gene Expression Analysis

The collected tobacco leaf samples were thoroughly ground in liquid nitrogen, and total RNA was extracted according to the manufacturer's protocol of the Plant RNA Extraction Kit. The RNA concentration and purity were measured using a NanoDrop 2000 (Thermo Fisher Scientific), and the quality and integrity of the total RNA were assessed by electrophoresis on a 1.0% agarose gel. First‐strand cDNA was synthesized from total RNA using a reverse transcription kit (Trans Gen Biotech, Beijing). The *NtGAPDH* gene was selected as an internal control. qRT‐PCR was performed in a 15 μL reaction volume containing 7.5 μL TB Green Master Mix, 2 μL cDNA template, 1 μL primer mixture (0.5 μL forward and 0.5 μL reverse primer), and 4.5 μL nuclease‐free water. The primers are listed in Table S12.

### 5.4 Subcellular Localization

For the leaf assay, *Agrobacterium tumefaciens* ( *A. tumefaciens* ) GV3101 strains carrying NtTAC1‐GFP, NtPIN3‐GFP, or the empty vector PC1300S‐GFP were generated by electroporation, cultured to logarithmic phase (OD_600_ = 0.6–1.0), and resuspended in infiltration buffer (10 mM MgCl_2_, 10 mM MES pH 5.6, 150 μM acetosyringone) to a final OD_600_ of 0.8–2. Each culture was then mixed with the appropriate marker (TAC1‐GFP) and the empty vector with the nuclear marker [OsGhd7‐mCherry], and NtPIN3‐GFP with the membrane marker [AtCBL1‐mCherry]. The mixtures were infiltrated into leaves of 4–5‐week‐old *Nicotiana benthamiana* plants. Fluorescence signals were examined by confocal microscopy 48 h post‐infiltration.

For the protoplast assay, protoplasts were isolated from three‐week‐old *Nicotiana benthamiana* leaves by enzymatic digestion, washed with W5 solution (154 mM NaCl, 125 mM CaCl_2_, 2 mM KH_2_PO_4_, 2 mM MES, 5 mM glucose, pH 5.7) and resuspended in MMG solution (0.4 M mannitol, 15 mM MgCl_2_, 4 mM MES, pH 5.7). NtTAC1‐GFP constructs, along with the empty vector control, were each co‐transfected with the nuclear marker nls‐mKATE into protoplasts with an equal volume of PEG4000 solution. After incubation at 25°C for 18–24 h, fluorescence signals were examined by confocal microscopy.

### 5.5 Detection of Phytohormones

For phytohormone quantification, the basal 1 cm segment of branches (immediately adjacent to the main stem) was excised from 10‐week‐old tobacco plants. Tissues from 10 plants were pooled as one biological replicate, with three replicates per genotype. Frozen leaf angle tissues (50 mg per sample) were ground and extracted with 1 mL methanol/water/formic acid (15:4:1, v/v/v) containing isotope‐labelled internal standards (10 μL, 100 ng/mL). After vortexing (10 min) and centrifugation (12 000 rpm, 5 min, 4°C), supernatants were dried under nitrogen, reconstituted in 100 μL 80% methanol, and filtered (0.22 μm). Analysis was performed using an ExionLC AD UPLC coupled to a QTRAP 6500+ MS. Separation used an ACQUITY UPLC HSS T3 C18 column (100 × 2.1 mm, 1.8 μm) at 40°C with a 0.35 mL/min flow rate. Mobile phases were water (A) and acetonitrile (B), each with 0.04% acetic acid. The gradient was: 0–1 min, 5% B; 1–8 min, 5%–95% B; 8–9 min, 95% B; 9–12 min, 5% B. Injection volume was 2 μL. MS detection used scheduled MRM mode with ion spray voltages of 5500 V (+) and −4500 V (−), source temperature 550°C, and curtain gas 35 psi. Optimized DP and CE were used for each analyte. Data were processed with Analyst 1.6.3 and MultiQuant 3.0.3. All solvents were HPLC grade. Authentic standards were stored at −20°C.

### 5.6 Lignin Determination

Lignin content was determined using a commercial assay kit (D799081, Sangon Biotech) according to the manufacturer's instructions. Approximately 50–100 mg of dried leaf tissue was ground into a fine powder and extracted using the buffer provided in the kit. Following the colorimetric reaction procedure, absorbance was measured at 280 nm using a spectrophotometer. Lignin content was calculated based on a standard curve and expressed as mg g^−1^ dry weight (DW). Three independent biological replicates were analysed for each sample.

### 5.7 Safranin‐Fast Green Staining of Plant Tissue Paraffin Sections

Plant tissue paraffin sections were deparaffinized in environmentally friendly dewaxing transparent liquid (G1128, Servicebio) for 20 min, followed by rehydration through a graded ethanol series (100% ethanol twice for 5 min each, then 75% ethanol for 5 min) and rinsed with running water. The sections were stained with Safranin O for 2 min and quickly rinsed with water to remove excess dye. Subsequently, the sections were briefly decolorized in a graded ethanol series (50%, 70%, and 80% ethanol for 3–8 s each) to adjust the staining intensity. The sections were then stained with Safranin O‐Fast Green (Plant Tissue) Staining solution (G1031, Servicebio) for 6–20 s and dehydrated again in three changes of anhydrous ethanol (5, 10, and 20 s, respectively). After clearing in xylene for 5 min, sections were mounted with neutral balsam. Images were captured using a light microscope.

### 5.8 Extraction of Single‐Nucleus Nuclei and snRNA‐Seq Library Construction

The experiment was performed under low‐temperature conditions to preserve RNA integrity. Leaf angle tissues were collected from 25 individual plants per genotype. Fresh or frozen tissue samples (leaf angle samples of WT and *NtTAC1*‐R) were rapidly dissected (< 0.5 cm^3^) and immersed in pre‐chilled nuclear isolation buffer (containing RNase inhibitor, DTT, and BSA). Frozen tissues were flash‐frozen on dry ice and stored at −80°C. Cell membranes were lysed using mechanical homogenization (Dounce homogenizer) combined with GEDIOR snRNA‐seq‐specific dissociation buffer to retain intact nuclei. The lysate was filtered through a 40‐μm cell strainer to remove debris, followed by centrifugation (500 × g, 5 min, 4°C) to enrich nuclei. Further purification was achieved via sucrose density gradient centrifugation or fluorescence‐activated cell sorting (FACS). Nuclear integrity was verified by Trypan Blue staining and microscopic examination, and concentration adjusted to 500–1000 nuclei/μL (CountStar automated cell counter). Purified nuclei were suspended in RNA‐stabilizing buffer, stored short‐term at −80°C. Library preparation was performed using the 10× Genomics Chromium Single Cell 3′ Reagent Kit.

### 5.9 Processing and Analysis of snRNA‐Seq Data

The 10× Genomics Cell Ranger Pipeline (v5.0.0) was employed to align sequencing reads from two snRNA‐seq libraries (control and invasion) against the Ntab‐K326 tobacco reference genome (https://solgenomics.net/ftp/genomes/Nicotiana_tabacum/sierro_et_al_2014/). Data preprocessing involved filtering out doublets from gel beads‐in‐emulsion with DoubletFinder (McGinnis et al. 2019) and using Seurat‐based quality by removing cells with unique molecular identifier (UMI) counts above 29 000, or those expressing fewer than 410 genes or more than 11 000 genes. Harmony (Korsunsky et al. 2019) was then used for batch effect correction at a resolution of 0.5. Uniform manifold approximation and projection (UMAP) visualization (Becht et al. 2019) and Pearson correlation analysis informed the cell cluster annotation, which was performed using the Plant Cell Marker (PCM) database (https://www.tobaccodb.org) and known marker genes for various cell types.

### 5.10 Construction of Co‐Expression Networks

High‐dimensional weighted gene co‐expression network analysis (hdWGCNA) (Morabito et al. 2023) was applied to infer the gene regulatory network responsive to *NtTAC1*. Only genes expressed in ≥ 5% of all analysed cells were included. In brief, we first aggregated data from 20 cells within the same cluster to generate pseudo‐cells for each cell type, and then constructed a gene co‐expression matrix. Genes that were closely co‐expressed were grouped into modules. The hub genes of each module were identified based on module eigengene connectivity (kME > 0.8) and statistical significance (*p* < 0.05). The randomForest package (Liaw and Wiener 2015) in R was utilized to evaluate the classification accuracy of the modules, and Cytoscape (v3.7.2) (Su et al. 2014) was employed to visualize the co‐expression networks of specific modules.

### 5.11 Pseudotime Trajectory Analysis

The DDRTree algorithm from Monocle 2 was utilized for unsupervised ordering (Trapnell et al. 2014) to construct a tree‐like structure and explore the differentiation trajectory of endodermal cells. Visualization of this trajectory was achieved with the plot_cell_trajectory and plot_pseudotime_heatmap functions. Additionally, GO and KEGG enrichment analyses were conducted to explore the functional characteristics of genes from different modules along the pseudotemporal order.

### 5.12 RNA In Situ Hybridization

Tissues were rinsed with PBS, immediately fixed in plant in situ hybridization fixative, dehydrated through a graded ethanol series, and embedded in wax. The paraffin sections were deparaffinized and treated with Proteinase K (20 μg/mL, Sangon Biotech, Shanghai, China). After rinsing with distilled water and washing three times with PBS (5 min each), the slides were pre‐hybridized with pre‐hybridization solution at 65°C for 25 min, followed by overnight hybridization with probe‐containing hybridization solution. Following stringent SSC washes, sections were incubated with the hybridization solution containing the probe (diluted 1:400) at 40°C for 3 h. Subsequently, they were incubated with normal rabbit serum as a blocking agent. Sections were blocked with normal rabbit serum for 30 min at room temperature. Then, sections were incubated with mouse anti‐digoxigenin‐labelled alkaline phosphatase (anti‐DIG‐AP) for 50 min at 40°C and then rinsed with TBS. Colour development was performed using BCIP/NBT substrate (AR1023, BOSTER). Blue‐purple signals indicate positive target gene expression, with red‐stained nuclei serving as reference. The RNA probes used in RNA in situ hybridization are provided in Table S13.

### 5.13 Vector Construction and Transformation

For RNAi vector construction, the interference fragment was amplified using reverse fragment primers and inserted into the pCambia2301‐KY‐RNAi vector via *Bam*HI/*Xba*I digestion and ligation. This vector contains the kanamycin resistance gene as a selectable marker. The ligation products were transformed into competent *Escherichia coli* DH5α cells, and positive clones were selected as intermediate carriers. The same interference fragment was then amplified using forward fragment primers and cloned into the intermediate carrier via *Kpn*I digestion and ligation. The resulting RNAi vectors, pCambia2301‐*NtPIN3*‐RNAi, were subsequently transformed into *A. tumefaciens* strain GV3101. The RNAi constructs were introduced into tobacco ( *Nicotiana tabacum* ) via *A. tumefaciens‐mediated* leaf disc transformation.

To construct a gene‐editing vector targeting the *SlTAC1* coding sequence in tomato, two target sites were designed, and corresponding PCR primers were synthesized (Table S12). The resulting PCR fragments were then digested with *Bsa*I and ligated into the pV58‐K vector, resulting in the final CRISPR expression construct. The genetic transformation procedure was initiated by introducing the plasmid vector into *A. tumefaciens* strain GV3101 via electroporation. Then, the CRISPR/Cas9 construct was introduced into tomato ( *Solanum lycopersicum* ) cv. Micro‐Tom via *A. tumefaciens* ‐mediated cotyledon transformation performed by Towin Biotechnology (Wuhan, China). Several T0 lines with *SlTAC1* loss‐of‐function mutations were successfully identified via PCR product sequencing.

### 5.14 Spatial‐Targeted Metabolome

Frozen leaf angle tissues were fixed with three drops of distilled water during sectioning, and then sectioned at 25 μm thickness using a Leica CM1950 cryostat at −20°C. The sections were mounted on indium tin oxide (ITO)‐coated electrically conductive slides and vacuum‐dried for 30 min. The dried tissue sections were sprayed with 3 mg/mL α‐cyano‐4‐hydroxycinnamic acid (CHCA) matrix dissolved in 70% acetonitrile/30% water using a matrix sprayer at 60°C, with a flow rate of 0.12 mL/min and pressure of 10 psi. The matrix was applied with 30 passes and 5 s of drying time between each pass.

MALDI timsTOF MSI was performed on a Bruker timsTOF flex mass spectrometer equipped with a 10 kHz smartbeam 3D laser. The laser power was set to 80% and fixed during analysis. Mass spectra were acquired in positive ion mode over a mass range of m/z 50–1300 Da, with a spatial resolution of 50 μm and 400 laser shots per spectrum. All mass spectra were normalized by root mean square (RMS). Further structural identification of metabolites was confirmed by MS/MS fragmentation on the same system.

### 5.15 Statistical Analyses

Data were analysed using GraphPad Prism 9 software. Statistical significance was determined by Student's *t*‐test or by one‐way ANOVA followed by Tukey's test. The exact number of biological replicates is indicated in the Figure legends.

## Author Contributions

L.W., J.G. and X.X. conceived and designed the experiments. L.W. drafted the manuscript. C.W., G.X., Z.M., S.W. and Z.L. participated in the main experiments in this work, with assistance from M.W. J.Z. performed data analysis. J.Y. and P.C. provided revisions to the scientific content of the manuscript. All authors contributed to the article and approved the submitted version.

## Funding

This work was supported by Beijing Life Science Academy, 2024200CD0170. Zhengzhou Tobacco Research Institute, China National Tobacco Corporation, 110202201001 [JY‐01], 110202001020 [JY‐03]. The Natural Science Foundation of Henan, China, 242300420179.

## Conflicts of Interest

Author Junping Gao, Zhen Ma, Shuaibin Wang were employed by China Tobacco Hunan Industrial Co. Ltd. The remaining authors declare that the research was conducted in the absence of any commercial or financial relationships that could be construed as a potential conflicts of interest. The authors declare no competing financial interest.

## Supporting information


**Figure S1:** Subcellular localization of NtTAC1‐GFP fusion protein in tobacco. (a) Agroinfiltration‐mediated transient transformation in *Nicotiana benthamiana* leaf. (b) PEG‐mediated transformation in *Nicotiana benthamiana* protoplasts.
**Figure S2:** Average gene number and UMI in all tobacco petiole base region cells identified by snRNA‐seq identification. *NtTAC1*‐R and WT represents the two.
**Figure S3:** Cluster correlation and cellular composition analysis by snRNA‐seq. (a) Correlation among all cell clusters (0–20). (b) Proportion of cells from each sample across identified clusters.
**Figure S4:** Identification of the top five specific up‐regulated differentially expressed genes (DEGs) within each cell cluster.
**Figure S5:** PCR‐positive detection of *SlTAC1* mutant transgenic plants.
**Figure S6:** Mutation type of *SlTAC1* in *SlTAC1* mutant (*SlTAC1*‐K) plants. Biallelic mutations were identified, with one allele carrying a 1‐bp deletion and the other allele carrying a 5‐bp deletion.
**Figure S7:** Relative expression levels of *NtPIN3* in various tissues of tobacco plants. Data are presented as mean ± SD (*n* = 3).
**Figure S8:** Relative expression levels of *NtPIN3* in WT and *NtPIN3*‐RNAi plants. Data are presented as mean ± SD (*n* = 3).

**Table S1:** Summary of the single cell sequencing dataset for NtTAC1‐RNAi and WT samples.
**Table S2:** Marker genes used for the identification of different cell types.
**Table S3:** KEGG enrichment of makergenes for each cell type.
**Table S4:** Differentially expressed genes (DEGs) for each cell type between the wild and NtTAC1‐RNAi samples.
**Table S5:** Gene ontology (GO) enrichment of differentially expressed genes (DEGs) for each cell type.
**Table S6:** KEGG enrichment of differentially expressed genes (DEGs) for each cell type.
**Table S7:** KEGG enrichment of differentially expressed genes (DEGs) for endodermis cell type.
**Table S8:** The gene number of all module.
**Table S9:** Accuracy of cell type classification assessed using Random Forest modelling.
**Table S10:** KEGG enrichment of differentially expressed genes (DEGs) for red module.
**Table S11:** KEGG enrichment of differentially expressed genes (DEGs) for midnightblue module.
**Table S12:** Primers used in this study.
**Table S13:** RNA probes used in RNA in situ hybridization.

## Data Availability

The data that support the findings of this study are openly available in China National Center for Bioinformation at https://ngdc.cncb.ac.cn/gsub/submit/gsa/list, reference number CRA029342.
