# Supplementary material for: Spatiotemporal Metabolome and Single‐Nucleus Transcriptome Integration Illuminates an Auxin Gradient Orchestrated by NtTAC1 Underlying Leaf Angle Regulation in Tobacco
Source: Plant Biotechnol J. 2026 Apr 27;24(8):4897–915. doi: 10.1111/pbi.70672 (PMC13387881; doi:10.1111/pbi.70672)
Supplement: Supplementary file 1 — Figure S1: Subcellular localization of NtTAC1‐GFP fusion protein in tobacco. (a) Agroinfiltration‐mediated transient transformation in Nicotiana benthamiana leaf. (b) PEG‐mediated transformation in Nicotiana benthamiana protoplasts. Figure S2: Average gene number and UMI in all tobacco petiole base region cells identified by snRNA‐seq identification. NtTAC1‐R and WT represents the two. Figure S3: Cluster correlation and cellular composition analysis by snRNA‐seq. (a) Correlation among all cell clusters (0–20). (b) Proportion of cells from each sample across identified clusters. Figure S4: Identification of the top five specific up‐regulated differentially expressed genes (DEGs) within each cell cluster. Figure S5: PCR‐positive detection of SlTAC1 mutant transgenic plants. Figure S6: Mutation type of SlTAC1 in SlTAC1 mutant (SlTAC1‐K) plants. Biallelic mutations were identified, with one allele carrying a 1‐bp deletion and the other allele carrying a 5‐bp deletion. Figure S7: Relative expression levels of NtPIN3 in various tissues of tobacco plants. Data are presented as mean ± SD (n = 3). Figure S8: Relative expression levels of NtPIN3 in WT and NtPIN3‐RNAi plants. Data are presented as mean ± SD (n = 3). [file PBI-24-4897-s002.docx]

**Supplementary Figures**


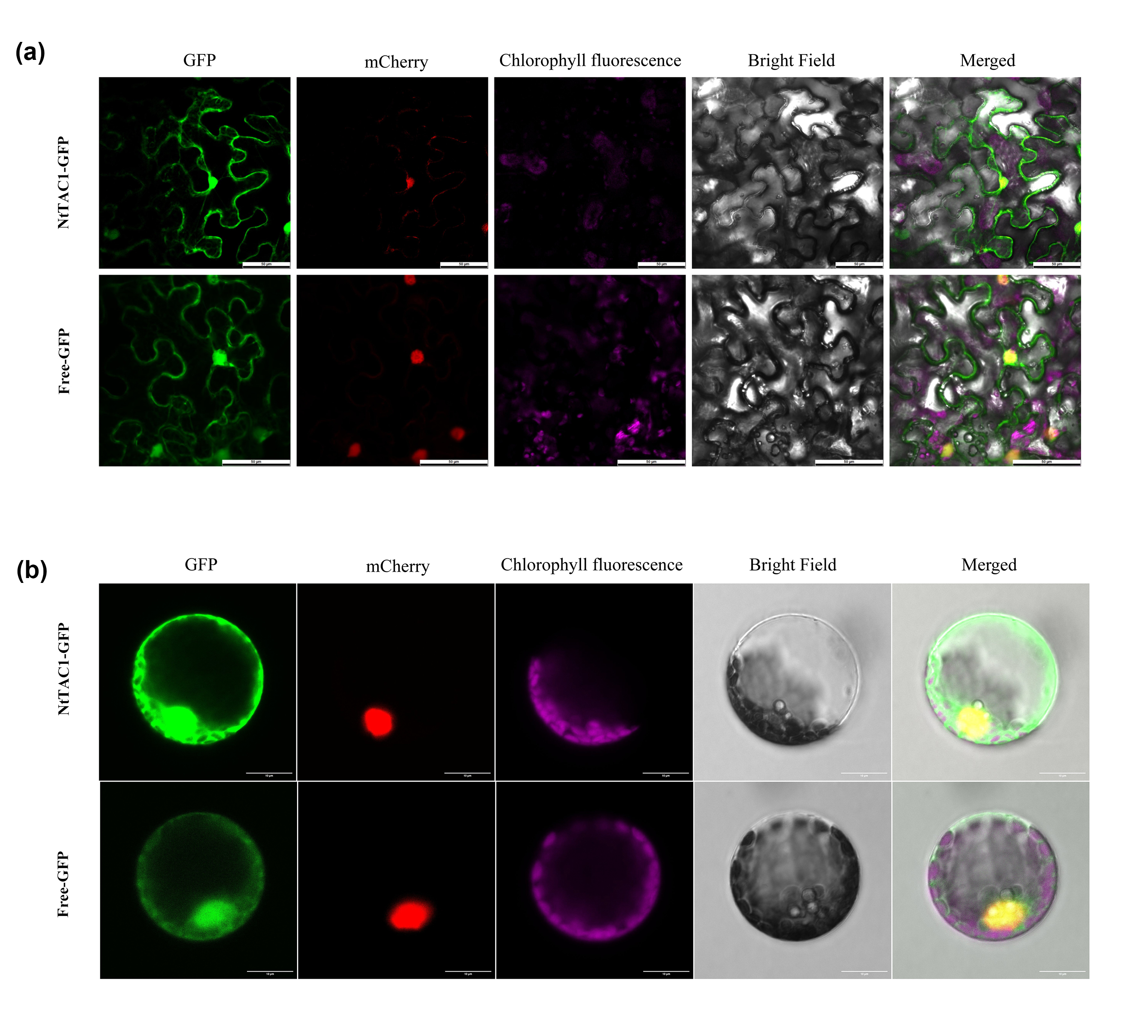


**Figure S1. Subcellular localization of NtTAC1-GFP fusion protein in tobacco.** (a) Agroinfiltration-mediated transient transformation in *Nicotiana benthamiana* leaf. (b) PEG-mediated transformation in *Nicotiana benthamiana* protoplasts.


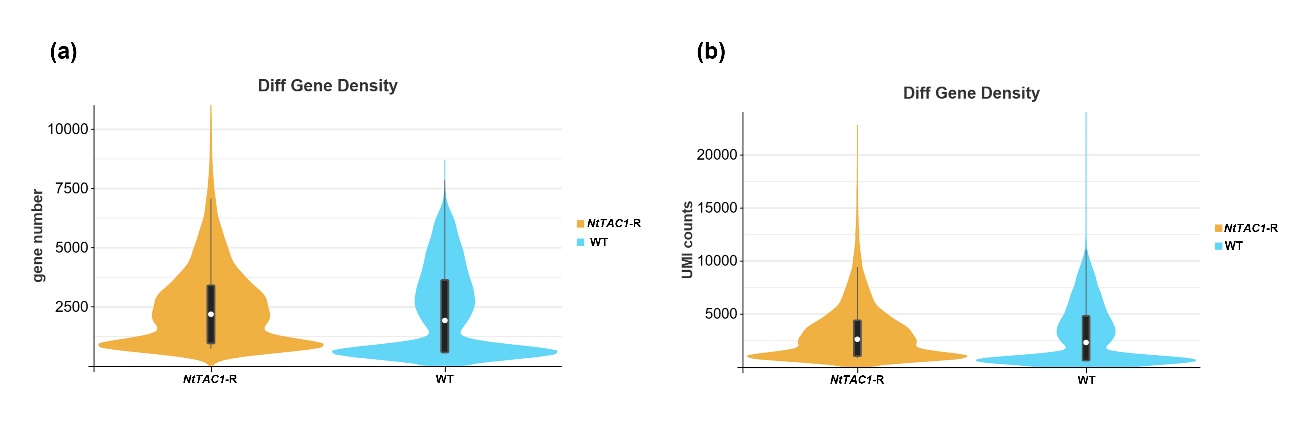


**Figure S2. Average gene number and UMI in all tobacco petiole base region cells identified by snRNA-seq identification.** *NtTAC1*-R and WT represents the two
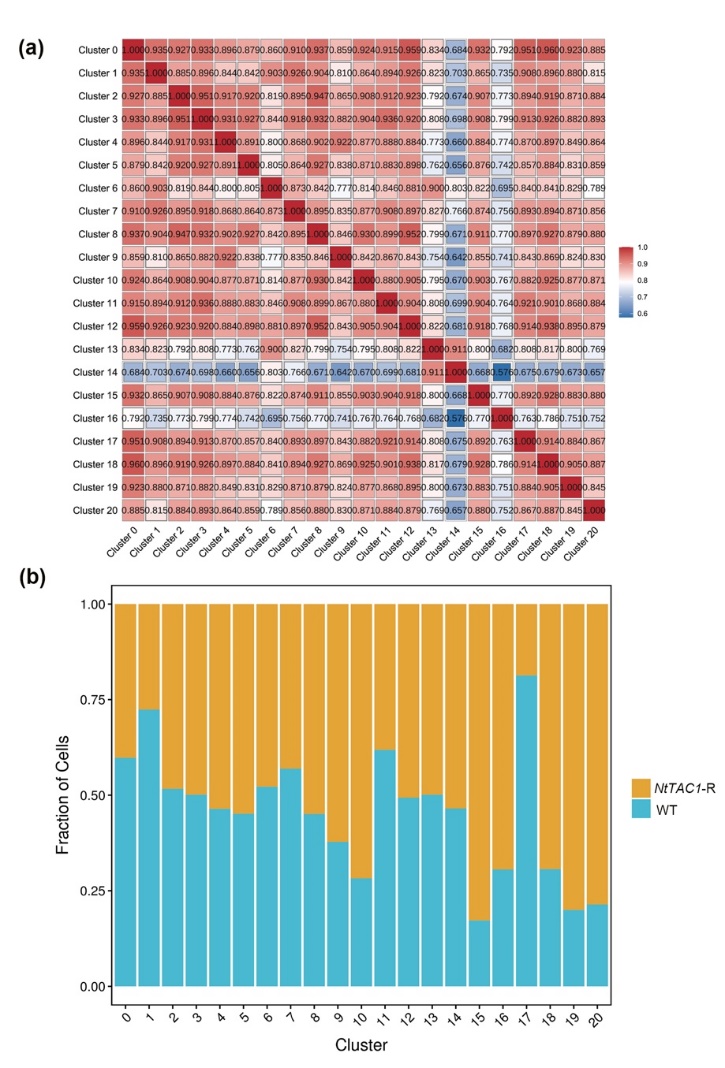


**Figure S3. Cluster correlation and cellular composition analysis by snRNA-seq.** (a) Correlation among all cell clusters (0-20). (b) Proportion of cells from each sample across identified clusters.

**
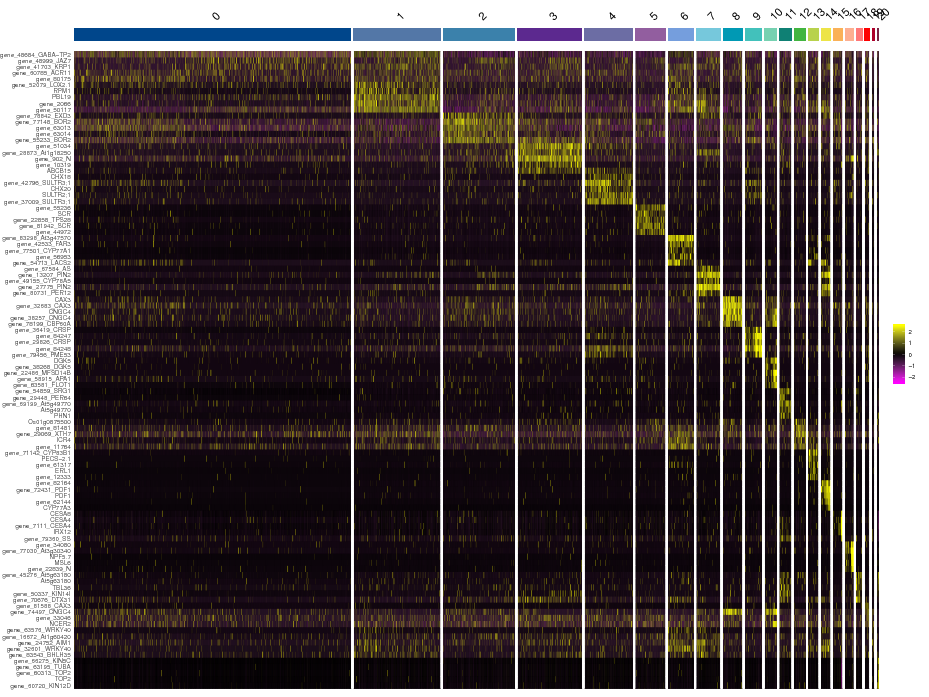
**

**Figure S4**. **Identification of the top five specific up-regulated differentially expressed genes (DEGs) within each cell cluster.**


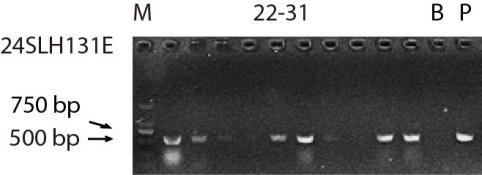


**Figure S5**. **PCR-positive detection of *SlTAC1* mutant transgenic plants**


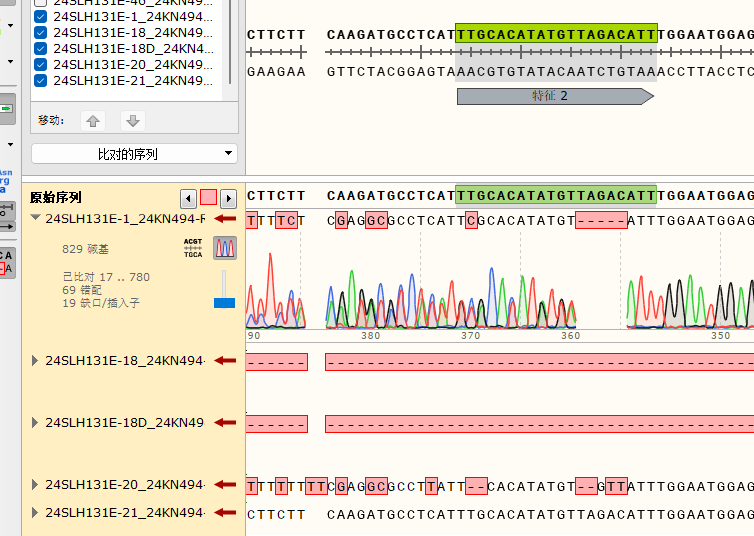


**Figure S6**. **Mutation type of *SlTAC1* in *SlTAC1* mutant (*SlTAC1*-K) plants.** Biallelic mutations were identified, with one allele carrying a 1-bp deletion and the other allele carrying a 5-bp deletion.


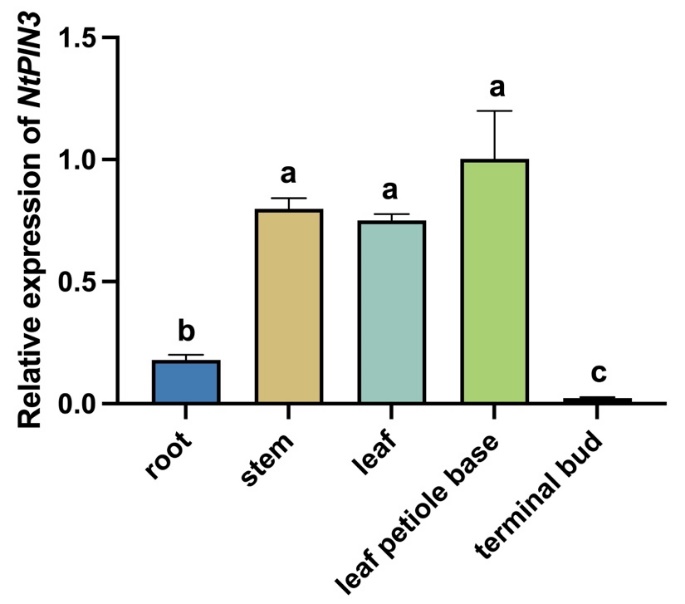


**Figure S7**. **Relative expression levels of *NtPIN3* in various tissues of tobacco plants.** Data are presented as mean ± SD (n = 3).

Statistical significance was determined by One‑way ANOVA followed by Tukey’s test. ***p* < 0.01, **p* < 0.05. n = biologically independent samples.


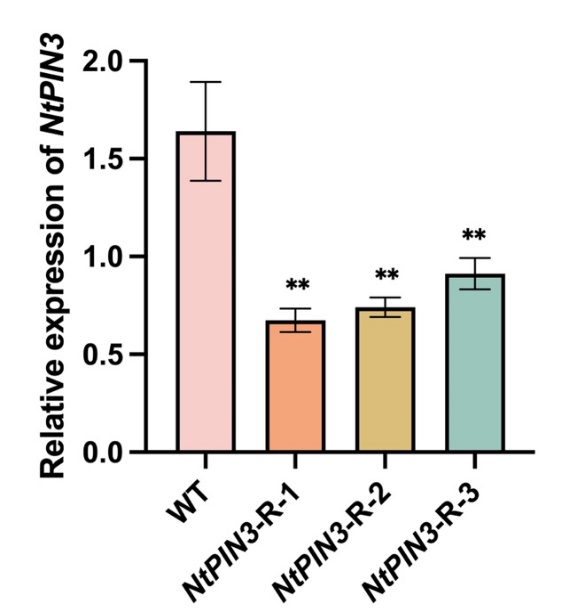


**Figure S8**. **Relative expression levels of *NtPIN3* in WT and** ***NtPIN3*-RNAi plants.** Data are presented as mean ± SD (n = 3).

Statistical significance was determined by Student's *t*-test. ***p* < 0.01, **p* < 0.05. n = biologically independent samples.
